# Integrated analysis of DNA methylation and mRNA expression profiles to identify key genes involved in the regrowth of clinically non-functioning pituitary adenoma

**DOI:** 10.18632/aging.102751

**Published:** 2020-02-03

**Authors:** Sen Cheng, Chuzhong Li, Weiyan Xie, Yazhou Miao, Jing Guo, Jichao Wang, Yazhuo Zhang

**Affiliations:** 1 Beijing Neurosurgical Institute, Capital Medical University, Beijing 100070, China; 2 Beijing Neurosurgical Institute, Beijing Tiantan Hospital Affiliated to Capital Medical University, Beijing Institute for Brain Disorders Brain Tumour Center, China National Clinical Research Center for Neurological Diseases, Key Laboratory of Central Nervous System Injury Research, Beijing 100070, China; 3 People's Hospital of Xin Jiang Uygur Autonomous Region, Urumqi 830001, China

**Keywords:** clinically non-functioning pituitary adenoma, DNA methylation, regrowth prediction

## Abstract

Tumour regrowth is a key characteristic of clinically non-functioning pituitary adenoma (NFPA). No applicable prognosis evaluation method is available for post-operative patients. We aimed to identify DNA methylation biomarkers that can facilitate prognosis evaluation. Genome-wide DNA methylation and mRNA microarray analyses were performed for tumour samples from 71 NFPA patients. Differentially expressed genes and methylated genes were identified based on the regrowth vs non-regrowth grouping. There were 139 genes that showed alterations in methylation status and expression level, and only 13 genes showed a negative correlation. The progression-free analysis found that *FAM90A1, ETS2, STAT6, MYT1L, ING2 and KCNK1* are related to tumour regrowth. A prognosis-prediction model was built based on all 13 genes from integrated analysis, and the 6-gene model achieved the best area under the receiver operating characteristic curves (AUC) of 0.820, compared with 0.785 and 0.568 for the 13-gene and 7-gene models, respectively. Our prognostic biomarkers were validated by pyrosequencing and RT-PCR. *FAM90A1* and *ING2* was found to be independent prognostic factors of tumour regrowth with univariate Cox regression. The DNA methylation and expression levels of *FAM90A1* and *ING2* are associated with tumour regrowth, and may serve as biomarkers for predicting the prognosis of patients with NFPA.

## INTRODUCTION

Pituitary adenoma (PA) is the third most common intracranial neoplasm [1]. PA arises from different anterior pituitary secretory cells and is therefore characterized by the over-secretion of certain hormones and causes relevant clinical symptoms. However, the largest subgroup of pituitary adenoma shows no specific serum hormone level change and mainly induces mass effects, including visual disturbance, headache and various degrees of hypopituitarism; this subgroup is called clinically non-functioning pituitary adenoma (NFPA) [2, 3].

Surgery has been suggested to be the most effective treatment for NFPA. However, approximately 12-58% of patients with macro-adenoma or giant adenoma will experience tumour regrowth within five years [4]. Compared with clinically functioning pituitary adenoma whose regrowth can be manifested by serum hormonal alteration, there is no practical approach to monitor the tumour regrowth of NFPA, except magnetic resonance imaging (MRI).

Interest in tumour epigenetic studies has attracted considerable attention in recent years. Epigenetic abnormalities, especially the aberrant DNA methylation, are recognized as hallmarks of tumorigenesis and patients' prognosis [5–10]. Studies have shown that DNA methylation may play a pivotal role in pituitary adenoma tumorigenesis, but the alteration in DNA methylation of tumour regrowth has not been fully illustrated yet [11].

In this study, we used whole-genome DNA methylation and mRNA microarray analysis and identified thirteen genes that showed differential DNA methylation and expression levels. We then established a regrowth prediction model with six genes that was also valuable in progress-free survival (PFS) analyses. Two genes were found to be independent prognostic factor of NFPA regrowth. We aimed to identify efficient DNA methylation and expression parameters to evaluate the regrowth of NFPA.

## RESULTS

### Identification of differentially methylated genes (DMGs)

**Figure 1:**
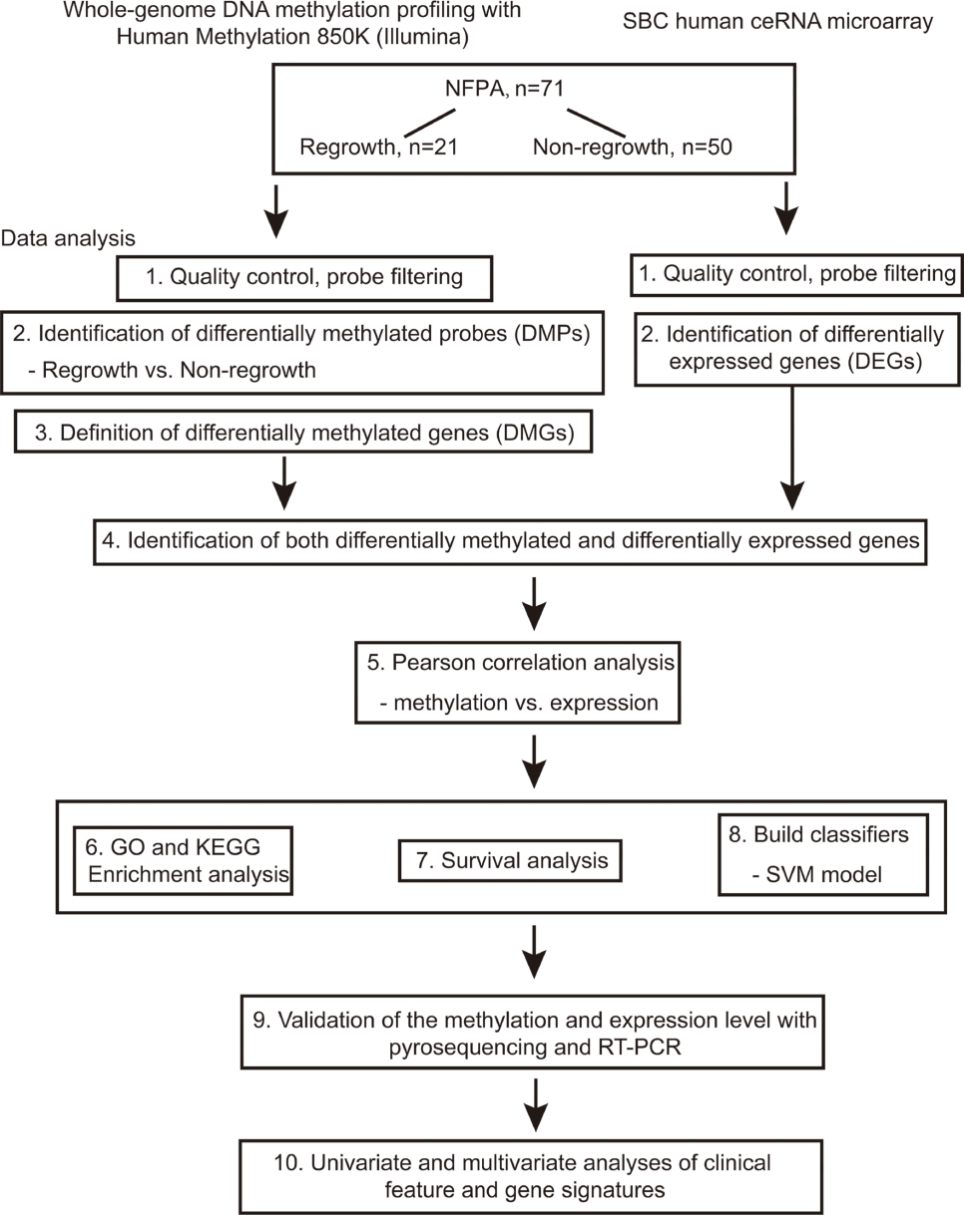
**Flowchart of this study.**

**Figure 2:**
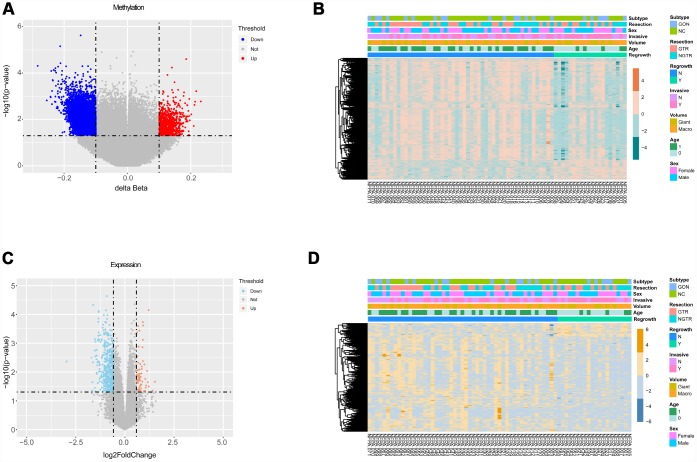
**Differential analyses of gene methylation and expression status between regrowth and non-regrowth patients.** (**A**) There are 3329 differentially methylated genes, of which 2788 hypomethylated genes (blue) and 541 hypermethylated genes (red) (**B**) The heatmap shows methylation profiles of 71 NFPA samples. The rows represent the different probes, and the columns represent each sample. The color in the heatmap represents the methylation level difference, which are hypermethylation (orange) and hypomethylation (green). The bar on the top shows the clinical and grouping information, and the sample ID is on the bottom. (**C**) The volcano plot shows 501 differentially expressed genes, and there are 438 upregulated genes(red) and 63 downregulated genes (blue). (**D**) The heatmap shows the expression profiles of the 71 NFPA samples. The rows represent the different genes, and the columns represent each sample. The color in the heatmap represents the expression level difference: upregulated (yellow) and downregulated (blue). The bar on the top shows the clinical and grouping information, and the sample ID is on the bottom.

The flowchart of the study is shown in Figure 1. Based on the criteria mentioned above, we divided all patients into regrowth and non-regrowth groups. The summary of clinical characteristics of these 71 patients (20 regrowth and 51 non-regrowth patients) are shown in Table 1 and detailed information is provided in Supplementary Table 1. We compared the DNA patterns in the two groups by high-throughput DNA methylation screening. After filtering the raw data and statistical analysis, we determined that 6636 CpG sites in promoter region which are related to 3329 different genes showing significant differences in promoter DNA methylation status between regrowth and non-regrowth patients, of which, 2788 genes were found to be hypomethylated and 541 genes were hypermethylated in regrowth group (Figure 2A and Supplementary Table 2). The heatmap showed the methylation status of these genes based on beta values of these CpG sites across all 71 samples (p < 0.05, |Δβ| > 0.1, Figure 2B).

**Table 1:** Clinical characteristics of 71 patients with NFPA.

	**N**	**Percentage**
**Gender**		
Male	36	50.7%
Female	35	49.3%
**Age**		
Mean	50.45±11.7	
Median	52	
**Tumour Volume**		
Macro-	51	71.8%
Giant	20	28.2%
**Invasive**		
Yes	39	54.9%
No	32	45.1%
**Subtype**		
Null cell	49	69.0%
Gonadotroph	22	31.0%
**Resection**		
GTR	35	49.3%
NGTR	36	50.7%

Abbreviations: Macro, macro-adenoma (1-4 cm); Giant, giant adenoma (>4cm); GTR, gross total resection; NGTR, non-gross total resection.

### Identification of differentially expressed genes (DEGs)

We measured the expression levels of 18854 genes between regrowth and non-regrowth patients. Five hundred one genes were differentially expressed (p < 0.05, fold change > 1.5 or < 0.67) between two groups, of which 63 genes were found to be upregulated and 438 genes were downregulated in regrowth group (Figure 2C and Supplementary Table 3). The heatmap and volcano plot of the DEGs are presented in Figure 2D.

### Integrated analysis of DMGs and DEGs

**Figure 3:**
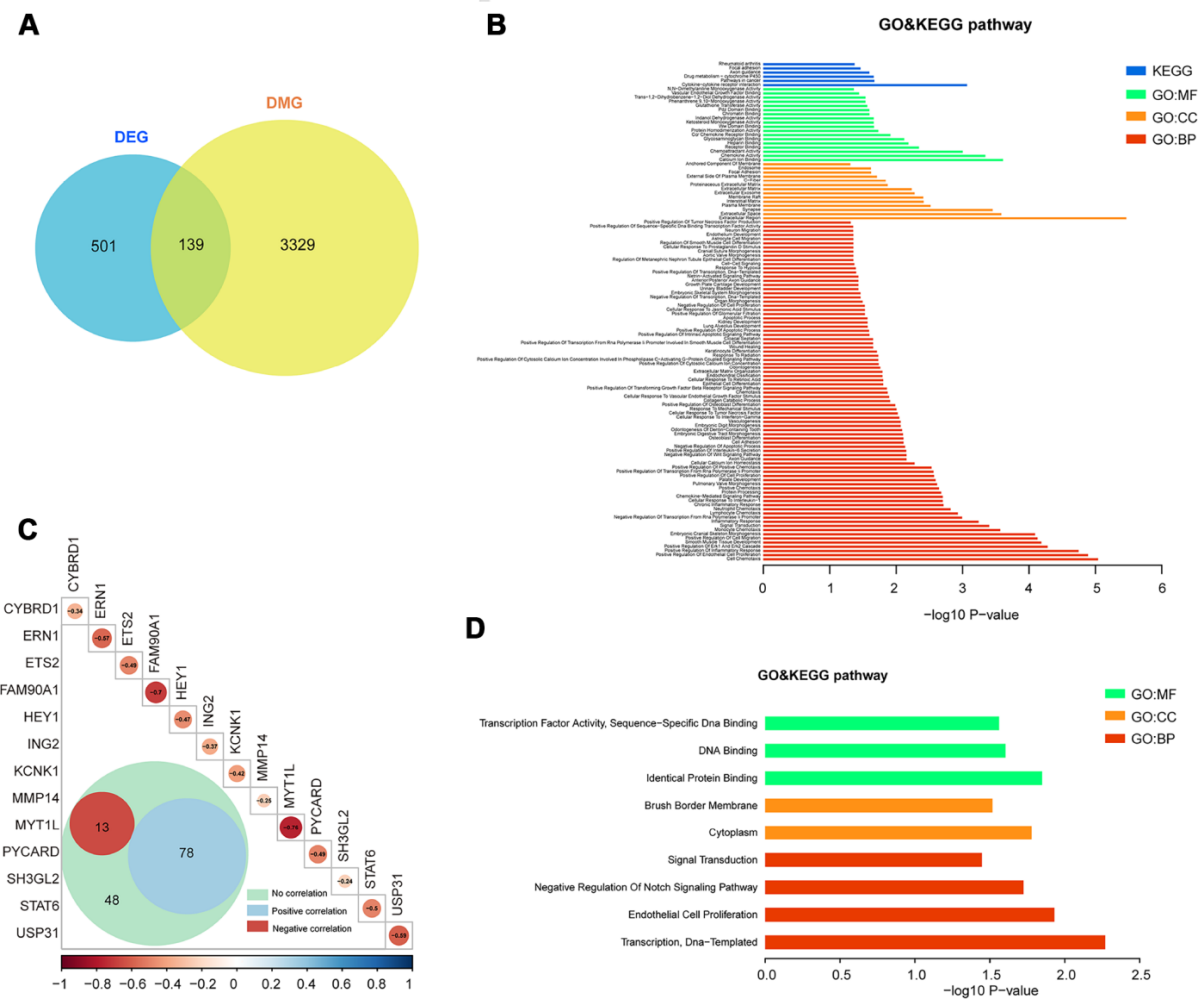
**Integrated analysis of DMGs and DEGs.** (**A**) The Venn diagram shows 139 genes with both DNA methylation and expression level changes. (**B**) GO and KEGG pathway analyses of 139 genes. (**C**) Pearson analysis of 139 genes. There are 13 genes showing negative correlation (red), 78 genes showing positive correlation (blue) and 48 genes showing no correlation. The R value of 13 genes is shown. (**D**) GO and KEGG pathway analyses of 13 negative correlation genes.

We only focused on genes that showed both expression changes and methylation alteration. To this end, 139 of the 501 genes are both differentially promoter methylated and expressed (Figure 3A). The enrichment analysis showed that these genes are tumour related, such as cell adhesion, apoptotic process, signal transduction, extracellular region, and pathways in cancer (Figure 3B and Supplementary Table 5).

We further studied the correlation of 139 genes’ promoter methylation status and expression state by Pearson correlation analysis. Then, 13 genes (cytochrome b reductase 1 [*CYBRD1*], endoplasmic reticulum-to-nucleus signaling 1 [*ERN1*], ETS proto-oncogene 2 [*ETS2*], family with sequence similarity 90 member A1 [*FAM90A1*], hes related family bHLH transcription factor with YRPW motif 1 [*HEY1*], inhibitor of growth family member 2 [*ING2*], potassium two pore domain channel subfamily K member 1 [*KCNK1*], matrix metallopeptidase 14 [*MMP14*], myelin transcription factor 1-like [*MYT1L*], PYD and CARD domain containing [*PYCARD*], SH3 domain containing GRB2-like 2, endophilin A1 [*SH3GL2*], signal transducer and activator of transcription 6 [*STAT6*], ubiquitin specific peptidase 31 [*USP31*]) with negative correlation between DNA methylation and expression level were selected (p < 0.05, R < -0.2, Figure 3C and Supplementary Table 4). Enrichment analysis of these 13 genes also showed the relevance of tumour oncogenesis. (Figure 3D and Supplementary Table 6).

### Discovering candidate regrowth biomarkers in NFPA

**Figure 4:**
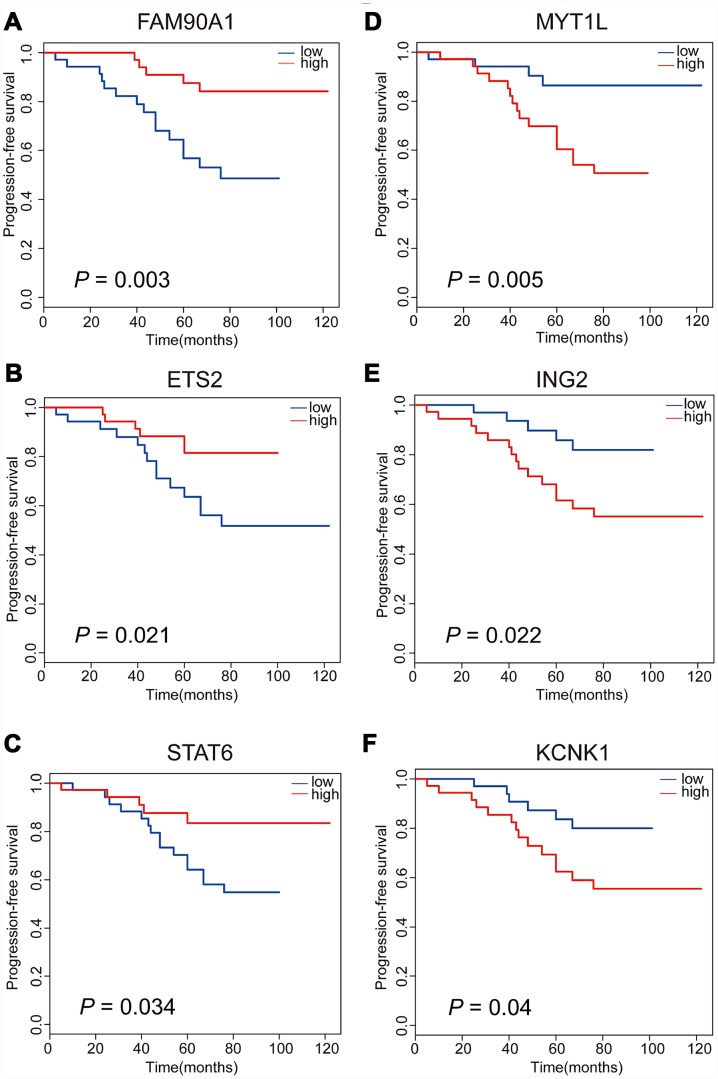
**Kaplan-Meier analyses of six significant genes in patients with NFPA.** Patients with upregulation of FAM90A1, ETS2 and STAT6 are less likely to have tumour regrowth (**A**–**C**). Patients with downregulation of MYT1L, ING2 and KCNK1 are less likely to have tumour regrowth (**D**–**F**).

Meanwhile, in order to assess the associations between gene expression level and time-to-event, we performed PFS analyses to further confirm whether there are possible candidates. The event endpoint was calculated from surgical resection to the date of the first regrowth. Patients were divided into 2 groups, a low-expression group and a high-expression group, according to the median cut-off of the gene expression levels. Our results indicated that patients with the over-expression of *FAM90A1, ETS2* and *STAT6* were less prone to face with regrowth, whereas the over-expression of *MYT1L, ING2* and *KCNK1* was correlated with poor prognosis (Figure 4). The expression levels of the other 7 genes were not significantly related to clinical outcome of NFPA (Supplementary Figure 1).

### Identification of potential regrowth predictive biomarkers in NFPA

**Figure 5:**
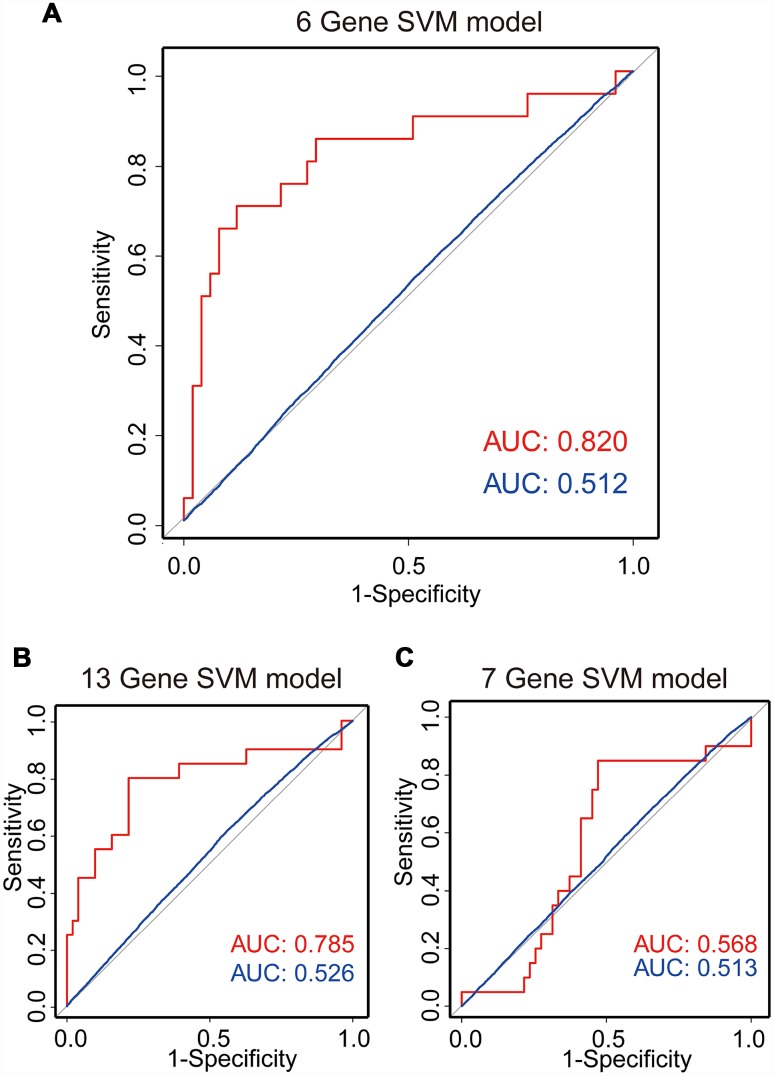
**SVM regrowth prediction model.** Three ROC curves using LOOCV show the comparisons of the AUC for the prediction of regrowth with 6 genes (**A**), 13 genes (**B**) and 7 genes (**C**). The red line shows the prediction model efficiency, and the blue line shows the permutation p-value of AUC was obtained from 1,000 randomization tests for testing the null hypothesis. The 6-gene model shows a better prediction accuracy.

Based on the above observations, these 6 of 13 genes (*FAM90A1, ETS2, STAT6 MYT1L, ING2* and *KCNK1*) were considered as potential biomarkers associated with the regrowth of NFPA. To further investigate whether these 6 genes could efficiently distinguish regrowth patients from non-regrowth patients, we performed unsupervised hierarchical clustering for 71 samples in the cohort according to the expression pattern of 6-gene biomarkers. We integrated these 6 genes to construct a 6-gene signature by developing a support vector machine (SVM) model. The performance of the model was evaluated in this cohort using the leave one out cross-validation (LOOCV) procedure, in which 70 samples were used as training set and the remaining one was served as the test sample. The results of the cohort showed that the model was able to correctly classify 20 out of 71 samples, achieving an overall predictive accuracy of 83.1%. The discriminatory performance of the model, evaluated by calculating the AUC, revealed that the AUC was 0.820, which was significantly higher than those of randomization tests (Figure 5A).

Meanwhile, we also construct a 13-gene signature and a 7-gene (except the 6 genes selected from 13 genes) signature by developing an SVM model. The performance of these models was evaluated in the same cohort using the LOOCV procedure. Our results showed that the 13-gene model was able to correctly classify 20 out of 71 samples, achieving an overall predictive accuracy of 80% with an AUC of 0.785, which were significantly higher than those of randomization tests (Figure 5B). Additionally, the 7-gene model was unable to correctly classify 20 out of 71 samples with an overall predictive accuracy of 59% with an AUC of 0.568, which were almost the same with those of randomization tests (Figure 5C). Our results demonstrated that the 6-gene model had better predictive performance for discriminating regrowth patients from non-regrowth patients.

### Validation of the DNA methylation and expression levels of the candidate predictive biomarkers

**Figure 6:**
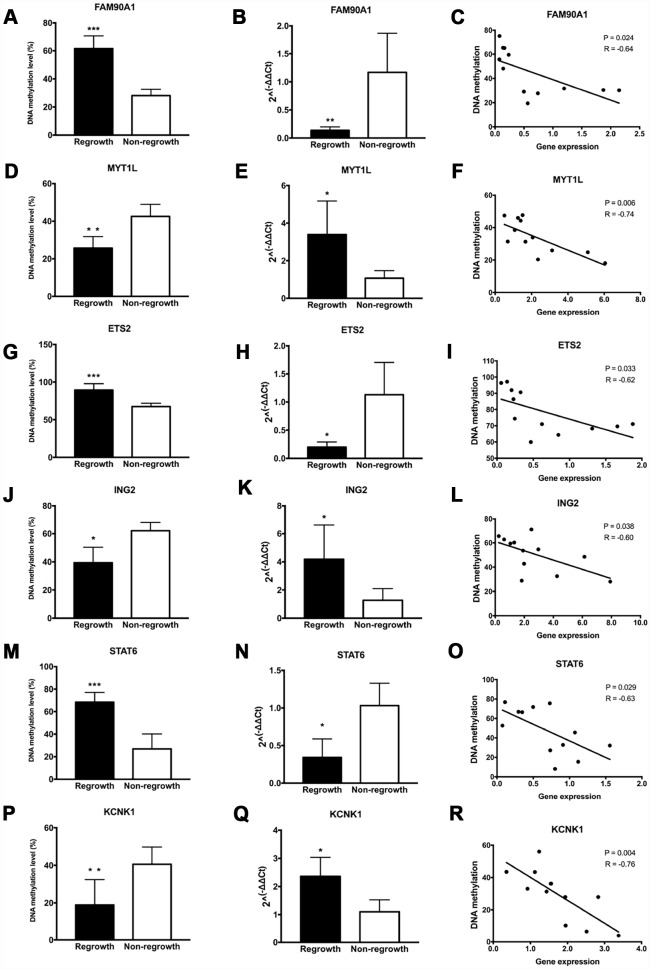
**Evaluation of DNA methylation and expression levels of selected genes.** The DNA methylation status, expression levels and Pearson correlation of FAM90A1, MYT1L, ETS2, ING2, STAT6, KCNK1 are shown. Each dot represents the average DNA methylation and gene expression level for every sample. * p < 0.05, ** p < 0.01, *** p < 0.001.

To further determine the authenticity of the DNA methylation level of the 13 gene signatures, pyrosequencing was performed in an additional set of patients based on the DMGs analyses. A significant increase of DNA methylation level in the regrowth group compared with non-regrowth group was observed in *FAM90A1, ETS2, STAT6, CYBRD1* and *PYCARD* (Figure 6A, 6G, 6M, Supplementary Figures 2A and 3A). Decreased methylation levels were observed in *MYT1L, ING2, KCNK1* and *SH3GL2* (Figure 6D, 6J, 6P and Supplementary Figure 3D). Conversely, no significant DNA methylation change was found in *ERN1, HEY1, MMP14, USP31* (Supplementary Figure 2C, 2E, 2G 2I).

Gene expression levels of 13 genes were assessed by RT-PCR in the same tumour samples that were used for pyrosequencing. We found a significant decrease in the expression level between the regrowth and non-regrowth groups in *FAM90A1, ETS2, STAT6, HEY1* and *PYCARD* (Figure 6B, 6H 6N, Supplementary Figures 2F and 3B). Increased expression levels were observed in *MYT1L, ING2, KCNK1, USP31* and *SH3GL2* (Figure 6E, 6K, 6Q, Supplementary Figures 2J and 3E). No significant expression change was found in *CYBRD1, ERN1* and *MMP14* (Supplementary Figure 2B, 2D, 2H).

Pearson analyses showed that the methylation status and expression levels of *FAM90A1, MYT1L, ETS2, ING2, STAT6, KCNK1, PYCARD, SH3GL2* showed a significantly negative correlation (Figure 6C, 6F, 6I, 6L, 6O, 6R and Supplementary Figure 3C, 3F).

These results confirmed that the methylation and expression levels of *FAM90A1, MYT1L, ETS2, ING2, STAT6* and *KCNK1* were consistent with the DNA methylation and mRNA microarray analyses.

### Clinical features and gene signatures related with tumour regrowth of NFPA

We used Cox regression analysis to identify the independent prognostic factors from clinical parameters and gene signatures. Through univariate and multivariate cox regression analyses, we found patients with younger age (HR = 0.323, 95% CI = 0.121 to 0.863, p = 0.024), decreased expression of *FAM90A1* (HR = 0.233, 95% CI = 0.083 to 0.649, p = 0.005) and increased expression of *ING2* (HR = 3.020, 95% CI = 1.067 to 8.543, p = 0.037) seems more likely to have tumour regrowth (Table 2). Age, expression of *FAM90A1* and *ING2* are independent prognostic factors of tumour regrowth. Our results showed that *FAM90A1* and *ING2* could be used as effective prognostic factors.

**Table 2:** Features related with tumour regrowth.

**Features**	**Univariate Cox regression**		**Multivariate Cox regression**	
	**HR (95%CI)**	**p value**	**HR (95%CI)**	**p value**
Gender (Male vs Female)	0.809 (0.328-1.992)	0.644		
Age (≥50 vs <50)	0.331 (0.126-0.872)	0.025	0.323 (0.121-0.863)	0.024
Volume (Giant vs Macro)	1.802 (0.648-5.013)	0.259		
Invasion (Invasive vs Non-invasive)	2.049 (0.804-5.222)	0.133		
Resection (Total vs Non-total)	0.898 (0.361-2.238)	0.818		
FAM90A1	0.278(0.100-0.775)	0.014	0.233(0.083-0.649)	0.005
MYT1L	3.023(1.088-8.395)	0.034	2.120(0.760-5.912)	0.151
ETS2	0.308(0.111-0.857)	0.024	0.526(0.173-1.596)	0.256
ING2	3.093(1.113-8.591)	0.03	3.020(1.067-8.543)	0.037
STAT6	0.411(0.148-1.140)	0.088		
KCNK1	1.936(0.762-4.919)	0.165		

Abbreviations: HR, hazard ratios; 95% CI, 95% confidence intervals; Macro, macro-adenoma; Giant, giant adenoma; Total, total resection; Non-total, non-total resection.

## DISCUSSION

Approximately 12%-58% patients with pituitary adenoma may face with tumour regrowth in 3-5 years [4]. For functioning pituitary adenoma, the changes in serum hormone levels and corresponding endocrine symptoms provide a feasible approach for regrowth evaluation. However, there is no specific endocrine symptoms in NFPA and it is mostly diagnosed and postoperatively monitored via imaging examinations. The chance of early intervention may have vanished when the symptoms of mass effect appear or when imaging examination shows tumour volume changes. For the above reasons, we attempt to identify efficient parameters to evaluate the regrowth of NFPA.

Although germline and somatic mutations are thought to be related to tumorigenesis of some types of pituitary adenoma, the drivers of NFPA is still unknown, as are the drivers of the regrowth of the tumour [12]. In the current field of NFPA, some genes have been reported to affect the prognosis of patients with NFPA and may be potentially used as prognostic biomarkers for NFPA. Ki-67 is commonly recognized as associated with regrowth of NFPA and high ki-67 index often indicates higher cell growth rate and shorter regrowth interval [13, 14]. Noh et al reported that high ki-67, PTTG1 as well as cell proliferation and apoptosis related genes, such as phospho-Akt, phospho-p44/42 MAPK, are related with tumour regrowth [15]. We have also tried to use known tumour-associated proteins and clinical factors to predict tumour regrowth and have obtained promising results [16]. However, above studies have not taken all molecular markers that may cause tumour regrowth into discussion, so some important markers may be missed. Therefore, in this study we performed genome-wide methylation and transcriptomic analysis, and tried to study the factors related to tumour growth from a broader perspective. Also, targeting DNA methylation sites as prognostic biomarkers for NFPA has not been explored, and our study is the first to report DNA methylation signatures combining genetic parameters for the prognosis evaluation of NFPA patients.

In this study, potential prognostic biomarkers of NFPA were found in sites of DNA methylation and expression in tumours of patients with NFPA. Thirteen genes that showed concordance in gene methylation status and expression levels were selected to perform the prognosis analysis and six genes were found to be related to the regrowth of NFPA. Through prognostic analyses of clinical parameters and six genes, age, *FAM90A1* and *ING2* was found to be the indenpendent factors of tumour regrowth. The DNA methylation and expression level of *FAM90A1* and *ING2* could serve as a new biomarkers for the early prognosis evaluation.

The use of Illumina Methylation 850K chip allowed a long list of aberrantly methylated CpGs to be identified. In combination with the mRNA microarray, we successfully recognized genes that may be regulated by DNA methylation. Several studies have identified that changes in DNA methylation status were correlated with poor pronosis of patients with pituitary adenoma [17–19]. In this study, we identified 2 genes which can be used as indenpedent risk factor of tumour regrowth. *FAM90A1* belongs to the family with sequence similarity 90 member, of which the encoding potential and fuction have not been fully illustrated but the variation in the organization and the number of copies of it is widely found in the human population [20, 21]. The methylation status of *FAM90A1* appeared to decrease mostly with age in postnatal development [22]. ETS2 interacts with Pit-1-binding within the PRL promoter. It is able to mediate transcriptional responses to growth factors and activate the Ras/mitogenactivated protein kinase (MAPK) pathway to influence the secretion of several different hormones in functioning pituitary adenoma [23]. The upregulation of ETS2 was found in acute myeloid leukaemia, prostate cancers, renal cell carcinoma and breast cancer, and it shows close correlation with poor prognosis [24–27]. In our study, we found that the methylation level of *ETS2* is higher in regrowth patients and that patients with upregulation of *ETS2* seem less likely to face with tumour regrowth. ING2 is a memeber of the inhibitor of growth family, of which represents as a tumour suppressor [28]. ING2 expression is usually decreased or lost in many human tumours, but another study found its expression to be upregulated [29–32]. However, The expression level and regulation mechanism has not been illustrated in pituitary adenoma. *KCNK1*, a member of the inwardly rectifying K+ channel family, encodes TWIK-1, which is a part of potassium (K^+^) channels [33, 34]. We found the KCNK1 gene upregulated in regrowth tumours, which may indicate a more active metabolism status. However, our results failed to illustrate the underlying mechanism within the methylation changes because we did not find the alterations of DNMT family, which are indispensable to *de novo* methylation and maintenance of methylation status [35, 36].

Clinical features such as age, gender, tumour volume and Knosp grade are widely accepted as factors that can affect the prognosis of patients with NFPA [4, 37–39]. The collection of information concerning these risk factors for NFPA regrowth is also of importance, which will provide valuable features and help to establish an effective prediction model. Because of limited sample size, number of patients in our study restricts us to evaluate the accuracy of our model in an additional validating set. However, our study is the first to try integrating DNA methylation signatures with genetic parameters for the prognosis of NFPA patients. Our further research intends to enlarge the patients' cohort and adjust our prediction model to integrate with clinical parameters for future clinical practice.

There is another limitation of our study. We realize the importance of cell-based experiment, but we did not perform cell-based studies except pyrosequencing and RT-PCR for the following reasons. Firstly, there are no targeted methylated or demethylated drugs that could interfere the specific methylation sites, so it is impossible to specifically alter the methylation level of a gene like we interfere the gene expression using lentivirus, shRNA or siRNA. Also, there is no human or murine cell lines of NFPA. So far, the cell lines that is commonly used for research are murine cell lines of functioning pituitary adenomas. Therefore, we did not explore their functions here. But, we are trying to establish the human cell line, and we will study the function of these genes in further research.

In conclusion, this is the first study to integrated DNA methylation and gene expression in NFPA in order to effectively identify key genes underlying tumour regrowth. The DNA methylation and expression levels of *FAM90A1* and *ING2* may serve as biomarkers for predicting the prognosis of patients with NFPA. Our study may promote the prognosis evaluation and early intervention to patients with NFPA.

## MATERIALS AND METHODS

### Patients

We retrospectively collected regrowth and non-regrowth NFPA samples from 71 patients diagnosed with NFPA at Beijing Tiantan Hospital from October 2007 to July 2016. All patients were performed enhanced head MRI scans before and after surgery to assess the maximum tumour diameter, Knosp grade and tumour resection rate. The criteria of regrowth were defined as maximum tumour diameter increases more than 2 mm on enhanced MRI from the day of surgery to follow-up endpoint with or without the reappearance of visual disturbance, headache or hypopituitarism. The minimum follow-up time was 17 months, and the average follow-up time was 64.9 months (range, 17-126 months).

### Tissue sample preparation

All samples were obtained from the Beijing Tiantan Hospital, neurosurgical department. Tumour samples were immediately put into liquid nitrogen and stored at -196°C once they were resected from the sellar region. A total of 71 pituitary adenoma samples were included to perform methylation and mRNA microarray analysis.

### Whole genome DNA methylation microarray

Total DNA was isolated and purified using the DNeasy Blood and Tissue Kit (Cat#69504, QIAGEN, Germany). The bisulfite conversion of the tumour DNA was performed using an EZ DNA Methylation-Gold™ Kit (D5005, Zymo, USA). Illumina Infinium MethylationEPIC 850K BeadChip was used to analyze the total DNA methylation status of 71 NFPA samples. The DNA methylation microarray experiment was performed following the manufacturer's instructions of Illumina at Shanghai Biotechnology Corporation. Probes located on the X and Y chromosomes, associated with a known SNP or bound to multiple genomic locations were excluded. We also removed probes that failed to be detected above background. The bio-conductor R package *minfi* (v1.18.6) was used for quality control and normalization of the raw data. The methylation status of a CpG site was calculated with the mean-difference β-value (Δβ) in promoter regions, which is between 0 (unmethylated) and 1 (completely methylated). DMGs that showed |Δβ| of 0.1 and adjusted p-value < 0.05 were defined as significantly differentially methylated.

### Whole-genome mRNA microarray

Total RNA was extracted and purified with the mirVana™ miRNA Isolation Kit (Cat#AM1561, Ambion, USA) and subsequently amplified and labeled with the Low Input Quick Amp WT Labelling Kit (Cat#5190-2943, Agilent Technologies, Santa Clara, CA, US), following the manufacturer’s instructions. RNA samples were then used to generate fluorescence-labelled cRNA targets for the SBC human ceRNA array V1.0 (4×180 K). The labelled cRNA targets were then hybridized and scanned on the Agilent Microarray Scanner (Agilent Technologies, USA). The data were extracted with Feature Extraction software 10.7 (Agilent Technologies, USA). Raw data were normalized by the Quantile algorithm using the *limma* package of R. The microarray experiments were performed according to the protocol of Agilent Technologies at Shanghai Biotechnology Corporation. Differential gene expression was determined using a t-test in the *limma* package. The fold change method was used to estimate the differential significance of genes. In this study, regrowth-related DEGs in 71 NFPA samples were defined as genes with an absolute fold change > 1.5 or < 0.67, and p-value < 0.05.

### Integrating analysis

We integrated DNA methylation and gene expression by a two-step analysis process. First, we identified differentially promoter methylated and expressed genes in 20 regrowth and 51 non-regrowth patients. Second, for both differentially promoter methylated and differentially expressed genes, we tested whether there is a strong association between DNA methylation and gene transcription. Pearson correlation analysis was used to assess the correlations. In this analysis, we applied the pearson correlation coefficient (PCC) and p-value to investigate the correlation and significance between mRNA expression and DNA methylation with the function cor.test in R. A negative correlation was considered significant if PCC < -0.2 and p-value < 0.05.

Functional enrichment analysis of Gene Ontology (GO) and Kyoto Encyclopedia of Genes and Genomes (KEGG) for target genes was performed to infer potential biological processes and pathways of regrowth-related genes using DAVID Bioinformatics Tool (version 6.8) [40]. The biological processes and pathways with a p-value < 0.05 using the whole human genome as a reference were considered as significant functional categories.

### Survival analysis

The PFS analysis in our study was defined as the time from the surgery to the first diagnosis of regrowth. Patients with loss of follow-up or study end date were considered censored in the survival comparison analysis. Survival outcomes were estimated using the Kaplan-Meier method, and differences were tested using the log-rank test, and a p-value < 0.05 was considered statistically significant.

### Development of regrowth related signature in NFPA

Candidate genes were integrated to form a regrowth-focus signature using a support vector machine with the sigmoid kernel to classify the regrowth and non-regrowth patients. An unbiased performance estimate in identifying regrowth patients was carried out using LOOCV. The diagnostic ability of the regrowth-focus prediction model was evaluated by obtaining the area under a receiver operating characteristic (ROC) curve. The ROC curve was produced by plotting true positive rates (sensitivity) against false positive rates (1-specificity). The permutation p-value of AUC was obtained from 1,000 randomization tests by randomizing mRNA expression data for testing the null hypothesis.

### Pyrosequencing assay

To validate the DNA methylation level of target genes in the promoter region, pyrosequencing assay was performed in another patient set (n = 12). The DNA methylation level of target genes in the promoter region was assessed by pyrosequencing assay. Total DNA from tumour samples was extracted with a QIAamp DNA Mini Kit (Qiagen, Germany). For each portion, 0.5 μg DNA was prepared to perform bisulfite conversion with EpiTect Bisulfite kit (Qiagen, Germany), according to the manufacturer's protocol. 1 μl of 10 μl eluted bisulfite converted DNA was prepared for PCR using a PyroMark PCR Kit (Qiagen, Germany), according to the manufacturer's instructions. The methylation level of the selected gene is measured as the percentage of average methylation in targeted CpG sites. The PCR primer used for the pyrosequencing assay was shown in Supplementary Table 7.

### Real-time quantitative reverse transcription polymerase chain reaction (RT-PCR)

Extraction and purification procedures of the tumour sample are the same as described above. Reverse transcription-PCR analyses were performed using the PrimerScript RT Reagent Kit (Takara, China). Quantitative real-time PCR was performed using LightCycler 480 SYBR Green I Master (Roche, Switzerland) with the ABI 9700 PCR system (Applied Biosystems, USA). The reaction steps were as follows: 95°C for 10 min followed by 40 cycles of 95°C for 10 s and 60°C for 30 s. The expression level was assessed by the standard curve method and normalized to the level of β-actin and was calculated using the 2^-ΔΔct^ method [41]. The primer sequences are shown in Supplementary Table 8.

### Statistical analysis

All statistical analyses and modelling were performed in R (version 3.1) and IBM SPSS Statistics for Windows, version 21.0. The clinical characteristics were analysed with univariate and multivariate Cox regression analyses. Hazard ratio (HR) was calculated using Cox proportional hazards regression. The differences were considered to be statistically significant for p < 0.05.

### Ethics approval

This study was approved by the medical ethics committee of Beijing Tiantan Hospital.

## Supplementary Material

Supplementary Figures

Supplementary Tables

Supplementary Table 1

Supplementary Table 2

Supplementary Table 3

Supplementary Table 4

Supplementary Table 5

Supplementary Table 6
