# Supplementary material for: Integrated analysis of DNA methylation and mRNA expression profiles to identify key genes involved in the regrowth of clinically non-functioning pituitary adenoma
Source: Aging (Albany NY). 2020 Feb 3;12(3):2408–27. doi: 10.18632/aging.102751 (PMC7041752; doi:10.18632/aging.102751)
Supplement: Supplementary Figures [file aging-12-102751-s008..pdf]

SUPPLEMENTARY FIGURES

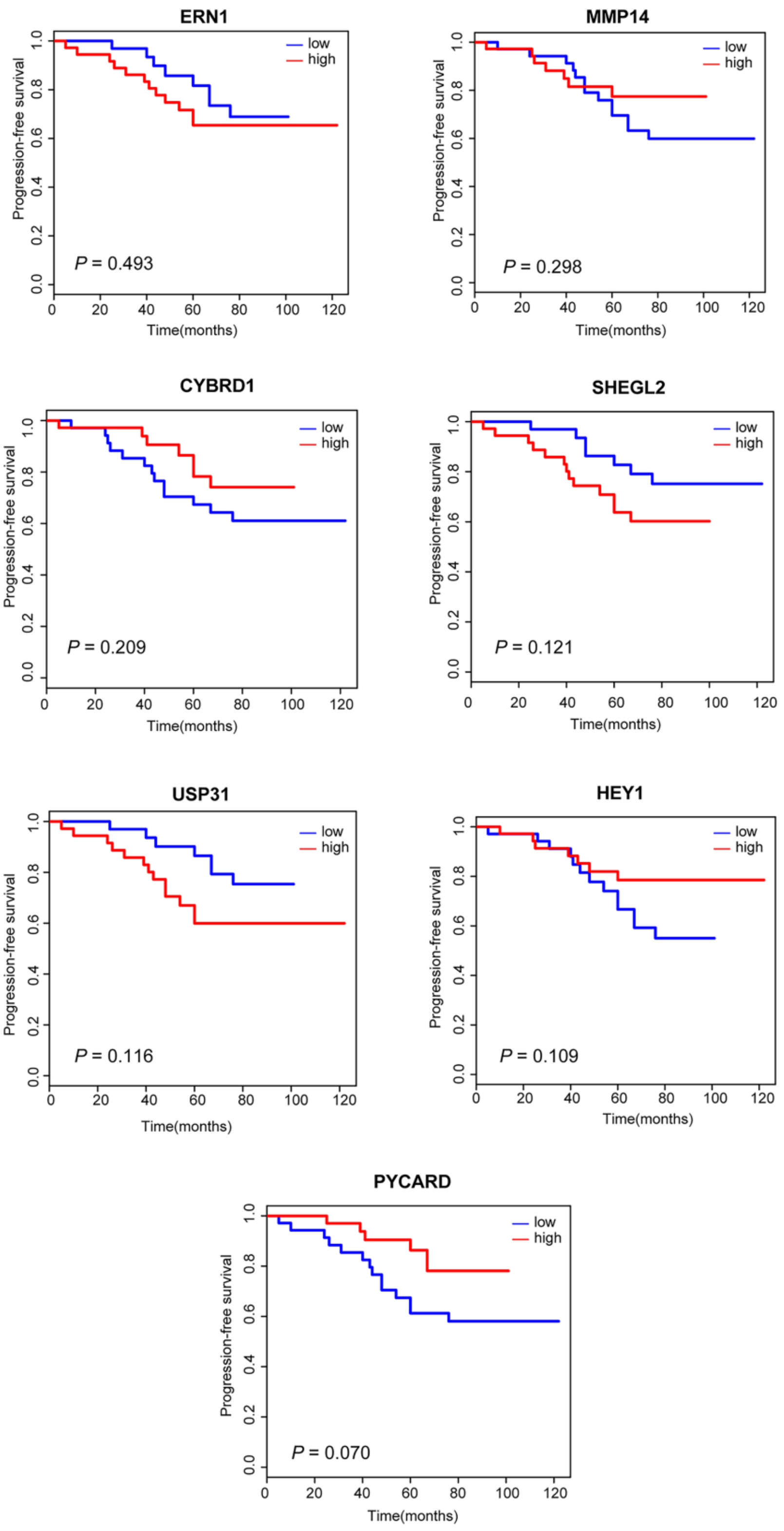

Supplementary Figure 1. Kaplan-Meier analyses of CYBRD1, ERN1, HEY1, MMP14 and PYCARD, in patients with NFPA.

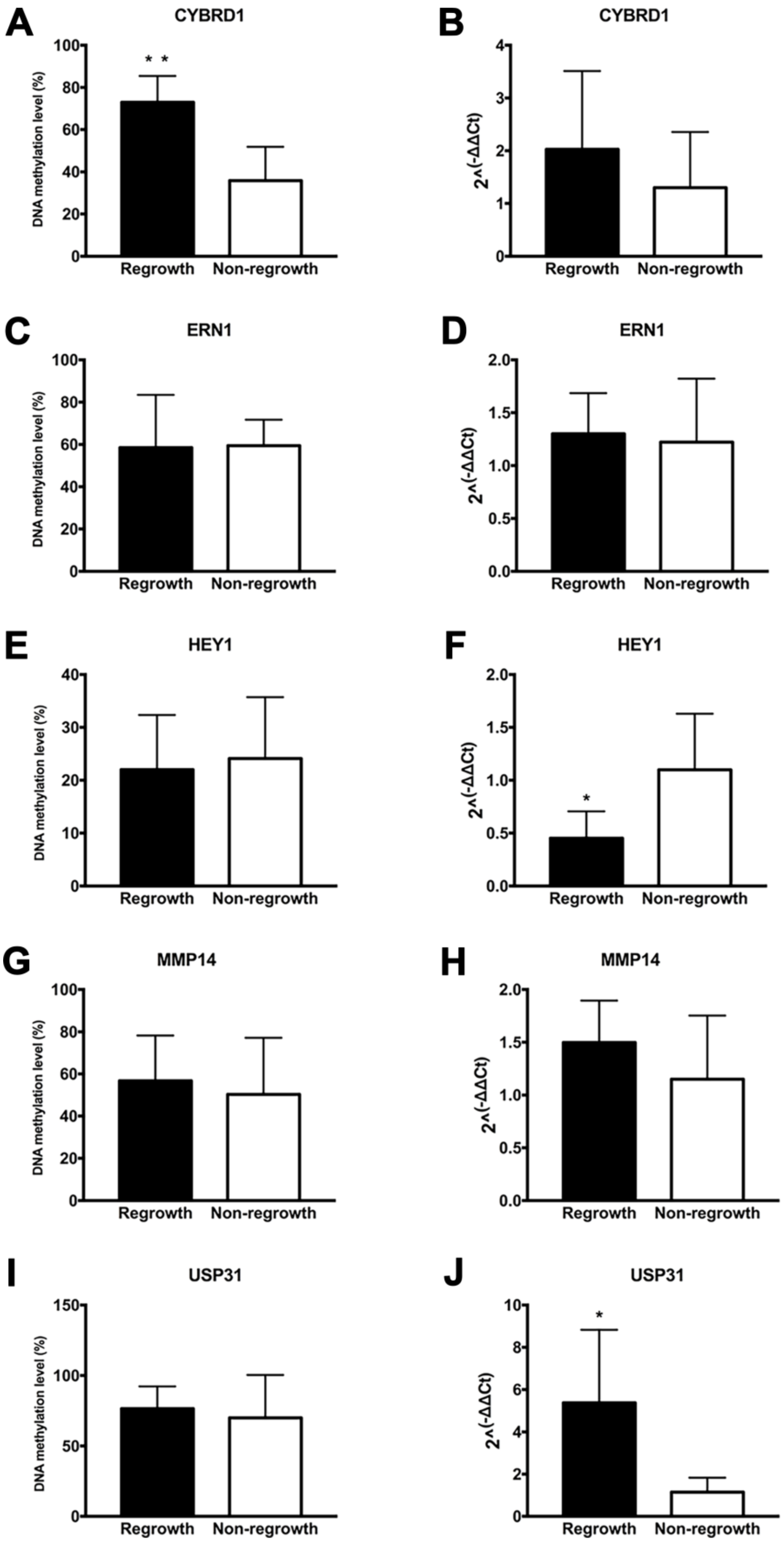

Supplementary Figure 2. The DNA methylation status and expression levels of CYBRD1, ERN1, HEY1, MMP14 and PYCARD. * $p < 0.05$, ** $p < 0.01$.

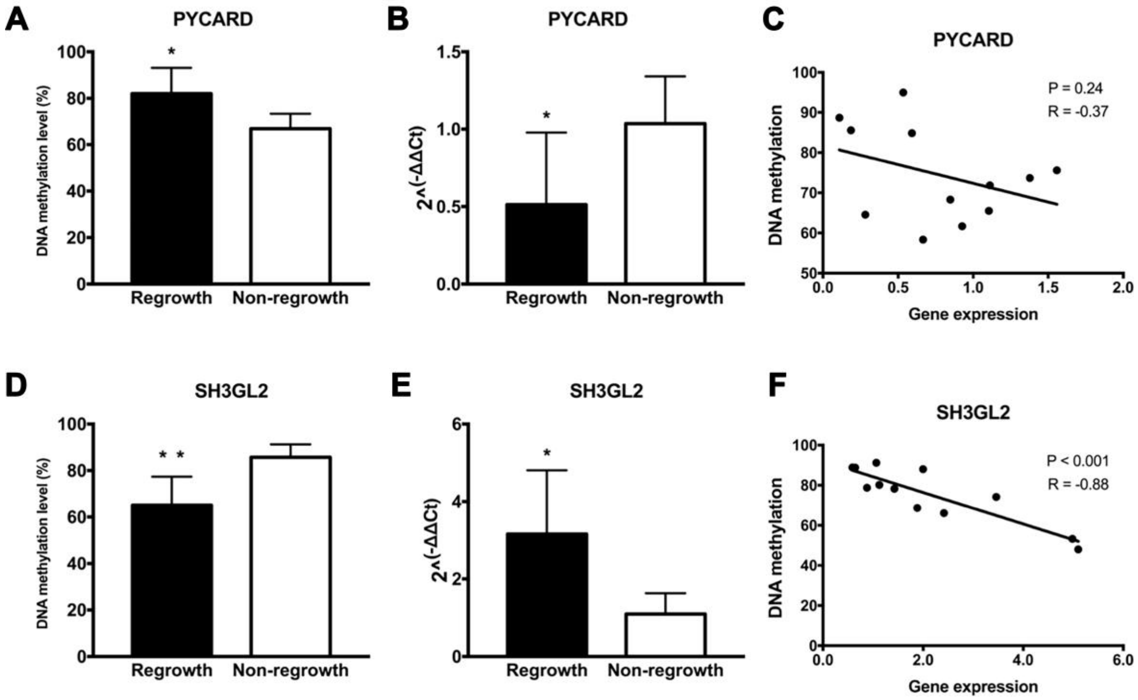

Supplementary Figure 3. The DNA methylation status, expression levels and Pearson correlation of SH3GL2 and USP31. Each dot represents average DNA methylation and gene expression level for every sample. * $p < 0.05$, ** $p < 0.01$.
