# Supplementary material for: Integrated analysis of DNA methylation and mRNA expression profiles to identify key genes involved in the regrowth of clinically non-functioning pituitary adenoma
Source: Aging (Albany NY). 2020 Feb 3;12(3):2408–27. doi: 10.18632/aging.102751 (PMC7041752; doi:10.18632/aging.102751)
Supplement: Supplementary Tables [file aging-12-102751-s007..pdf]

SUPPLEMENTARY TABLES

Please browse Full Text version to see the data of Supplementary Tables 1 to 6.

Supplementary Table 1. Clinical information of 71 patients with NFPA.

Supplementary Table 2. List of differentially methylated genes.

Supplementary Table 3. List of differentially expressed genes.

Supplementary Table 4. Pearson analyses of 139 genes.

Supplementary Table 5. GO and KEGG analyses of 139 genes.

Supplementary Table 6. GO and KEGG analyses of 13 genes.

Supplementary Table 7. Pyrosequencing primers of 13 genes.

No.	Gene	Forward primer	Reverse primer	Sequencing
1	FAM90A1	GGGTTAGGATGGGGTTTGATTGGA	TCCCTCAAAAACCCTTTACCTTCAC	TTTGGGTTTGGGGTTA
2	MYT1L	GTGTTTTAATAAGATTGTAGGGAAAAGTT	CCATCATCATTATCTATAAAACCATCTATT	GGGTAGGAATGATAAATGTA
3	ETS2	AGGGTAAAGTGGAGATTAAATTTTAGTTGA	ATCCATTCCTCATCATTCATATTCT	TGAGTTTTTTATATAGTTTTTTGTG
4	ING2	GTGAAAGGAAATATGTGTTTTTAAAGAA	ACAATAACTCCATCTTAACTCCCTACAAC	AAAAATTTGTAAGTTTGTTGG
5	STAT6	GTGGTTTAGAAGAGGGGGAATTTT	ATCTCAACCCCTATCTACCC	TGTTGTAGAAGTTGAGATTTTTTAG
6	KCNK1	ATGTTTTAGGGTTAGGTTATTGTGTTAG	TCTTTCAAATTCCTCCCACTATCC	TGAATGAAGGTAGAATTTTTTTAGT
7	CYBRD1	AGTGTTAAAAGTAAGTGGTGAGTA	TTACCTAATAACCTCACCCTCTCC	ATTAGGTTTGTTATTGAAGTT
8	ERN1	AGTTTGATGTTTGTGGGGATTAATTAG	ATCCTCAATACTCACTTTTCTCTACA	GTTTTTTGGAAATTAGTGTG
9	HEY1	TGATTTGGTATAAGTTTTTTTGGGGAATAA	ACCTTCTAAATCTTCCCCTCAT	AAAGTAAAATTAGTTGTTTTTATA
10	MMP14	TGGGTATTTAGAAGAGAGTAGTATTAATT	TTACCCTCCTATCCTCCCCAAAAA	GGGGTAGTTAGGTTTTAG
11	PYCARD	GTTTGTTTGTTTTTGGGTTTGTATTAG	CTCTCTACCCTTTTATACAACTCATTTT	ATAAAAACCAACCCAAAT
12	SH3GL2	GGGTTTTAGTAATTAAAAAGAAATGATTAT	CCTTAATCAATATATCCCTCAACATTC	GTGTAGGTTAGGTATAGTT
13	USP31	GATATTTTTTTGAGGTTTATTTGAGATAGT	ACTATATATCAATCTACCCTATCTATTCC	GTAATTTTTTTTGTTTTAATAAATA

Supplementary Table 8. RT-PCR primers of 14 genes (including control).

No.	Gene	Forward primer	Reverse primer	Product length(bp)	Ta(°C)
1	FAM90A1	TGAGAGCAGTGTTTCGGAC	ACACACCTATTTACAAAGGGAT	107	60
2	MYT1L	GCCACCTAAAGATGGAGAAAT	CTCTAAGCTCTACTCCTGGTC	91	60
3	ETS2	ATAAACAGACATGTGACTGGG	CGTGGTTCAACCTCACATAC	81	60
4	ING2	CTGATATTTGAAGGGTAAGCAT	GAACTAGTCAATCTGCTGCC	89	60
5	STAT6	GCCAGCTTCCCTTCACTC	ATGGCTTAGGATCTATGACCC	67	60
6	KCNK1	AAAGTACATTGTGATTTGCAGC	CTGTTCATTTAGGGATTCGTG	71	60
7	CYBRD1	CTGATTGACCACAGTTGCC	TACAGTTTCCTGCTGGAGTTA	129	60
8	ERN1	TAGGCTGAGATGCACCAAG	CACCCTCTGAACTCCGTC	92	60
9	HEY1	AGCAGGTGTAGTTAAACGAC	ACAACACATCAGTACAACAGAG	63	60
10	MMP14	GAGAGTGAGACCCAGTGGA	CAGAAATCTAGCCGAACTGC	107	60
11	PYCARD	TAAGGGAGTCCCAGTCCTAC	AGGATGATTTGGTGGGATTG	102	60
12	SH3GL2	ATCATGTGGAGTGAAAGGC	ATTTATTGGAAACAGCGATGC	79	60
13	USP31	GGATAGTGATTTCTGTGTCGT	CTAGGAAAGACATCCTGCC	70	60
14	β-actin	CATTCCAAATATGAGATGCGTT	TACACGAAAGCAATGCTATCAC	133	60
